# Cellular Glucose Uptake During Breath-Hold Diving in Experienced Male Breath-Hold Divers

**DOI:** 10.1186/s40798-018-0126-3

**Published:** 2018-03-27

**Authors:** Nicola Sponsiello, Danilo Cialoni, Massimo Pieri, Alessandro Marroni

**Affiliations:** 1 Apnea Academy Research, Padova, Italy; 2 DAN Europe Research Division, Contrada Padune 11, 64026 Roseto degli Abruzzi, TE Italy

**Keywords:** Breath-hold diving, Glucose metabolism, Insulin metabolism, Hypoxia

## Abstract

**Background:**

The physiological and pathophysiological mechanisms that govern diving, both self-contained underwater breathing apparatus (SCUBA) and breath-hold diving (BH-diving), are in large part well known, even if there are still many unknown aspects, in particular about cell metabolism during BH-diving.

The scope of this study was to investigate changes in glycemia, insulinemia, and the catecholamine response to BH-diving, to better understand if the insulin-stimulated glucose uptake mechanism is involved in cellular metabolism in this sport.

**Methods:**

Twenty male experienced healthy breath-hold divers were studied. Anthropometric information was obtained. Glycemia, insulinemia, and catecholamine response were investigated before and after the series of BH-diving.

**Results:**

We found a statistically significant decrease in the blood glucose levels between before and after dives (mean 94.3 ± 11.6 vs. 83.5 ± 12.5 mg/dl) *P* = 0.001 and a statistically significant increase in blood insulin value (median 4.5 range 3.4/6.4 vs. 7.0 range 4.2/10.2 mcgU/ml) *P* < 0.0001. Also, we found a statistically significant increase of catecholamine production (median 14.0 range 8/18 vs. 15.5 range 10.0/21.0 μg) *P* < 0.0001.

**Conclusions:**

The increase in blood insulin during BH-diving associated with the decrease of blood glucose levels could indicate that the upregulating cellular uptake is not caused by activation of the specific glucose transporters.

Particular diving-related conditions such as the diving reflex, the intermittent hypoxia/hyperoxia, and the particular environmental condition could play an important role in the mechanism involved in glycemia decrease in BH-diving.

Our data confirm that the adaptations to BH-diving are caused by complex mechanisms and involve many peculiar responses still in large part unknown.

**Electronic supplementary material:**

The online version of this article (10.1186/s40798-018-0126-3) contains supplementary material, which is available to authorized users.

## Key Points

- This is the first systematic protocol that shows an increase of insulin blood value during breath-hold diving associated with the decrease of blood glucose levels.
- Our data suggest that the upregulating cellular uptake in breath-hold diving is not caused by activation of the specific glucose transporters.
- Our data confirm that the adaptations to BH-diving are caused by complex mechanisms and involve many unknown responses.

## Background

The popularity of self-contained underwater breathing apparatus (SCUBA) and breath-hold diving (BH-diving) is steadily increasing [1], and the physiological and pathophysiological mechanisms that govern these activities are in large part well known [2], even if cellular metabolism during BH-diving is not [3].

It is well known that the secretion of insulin does not increase during exercise in normobaric conditions [4] and that muscle contraction activates glucose insulin-independent mechanisms [5]. In fact, the increase in muscle glucose uptake during exercise occurs even if insulin levels were prevented from rising, in vitro and in vivo [6].

Several authors demonstrated that during exercise, an increase in specific glucose transporters assures an increase in glucose uptake. Muscle-specific glucose transporter type 4 (GLUT4) plays an important role in the transport of glucose inside the cells when the mechanism is insulin-independent [4, 6]. This mechanism is responsible for the reduction of glycemia during exercise when the insulin does not increase [7].

On the other hand, it is also well known that the decrease of blood glucose levels in cases of intermittent hypoxia is linked to an increase of insulin-stimulated glucose uptake both in vitro [8] and in vivo [9]. Clanton also has confirmed that glucose cellular uptake increases because hypoxia induces upregulation of hypoxia-inducible transcription factor 1 (HIF-1) [9]. In this condition, an increase in glycolysis and a decrease in the number of myocytes have been demonstrated [10].

The scope of this study was to investigate changes in glycemia, insulinemia, and catecholamine responses in BH-diving with the objective of better understanding if the insulin-stimulated glucose uptake mechanism is involved in cellular metabolism in BH-diving.

## Methods

### Subjects

Twenty healthy male experienced BH-divers were studied. Mean age was 34.1 ± 7.4, mean height 174.1 cm ± 6.3, mean weight 73.3 kg ± 10.5, and BMI 24.2 ± 3.1.

All subjects were affiliated to the “Apnea Academy” Training Agency as instructors:

This was a selected group of very skilled free divers with a common high level of specific preparation.

All the instructors met the minimum requirements to be admitted to the Apnea Academy instructor level:

- Minimum depth with constant weight: − 30 m;
- Minimum time of static apnea (at surface): 4 min;
- Minimum dynamic BH-diving in swimming pool (distance): 75 m

No subject had historical or clinical evidence of arterial hypertension and cardiac, pulmonary, or any other significant disease.

Divers made a series of five dives to 20 m with free recovery time at the surface and 5 s of static apnea at the bottom. No diet control before diving was given in this study.

Depth, diving time, bottom time, and surface interval were recorded for each dive, using a free-diving computer (UP-X1 Omersub, Spa, Monza Brianza, Italy). This computer measured and recorded diving data every 2 s and allowed for calculation of the maximum gradient factor (GF) according to the Buhlmann ZHL16 C model.

GF is a way to measure nitrogen supersaturation in the “leading tissue” at any given time and depth during the ascent to the surface.

Individual differences in the dive profiles dived by the 20 divers were recorded for further investigation.

Blood glucose level was measured immediately before and after the dive series by collecting a capillary blood sample using a commercial device (Accu-Check AVIVA, Roche Diabetes Care Italy S.p.A.Viale G.B. Stucchi 110-20900 Monza MI).

Blood insulin level was investigated by immunoassay technique.

Differences in glycemia and insulin were investigated before and after the dive series.

Catecholamines were investigated by collecting all urine produced from the start of the dive series until the first micturition after diving and compared with a comparable sample the day after diving.

### Statistical Analysis

Data are presented as the mean ± standard deviation (SD) for parametric data and median and range for non-parametric data. Differences between before and after the BH-diving series were investigated using the Mann-Whitney *U* test after Kolmogorov-Smirnov normality test for non-parametric data and two-sample *t* test for parametric data. Differences in dive profile between the 20 divers were investigated using non-parametric repeated measures (analysis of variance ANOVA Kruskal-Wallis test), followed by Dunn’s post hoc multiple comparison tests.

A probability lower than 5% was assumed as the threshold to reject the null hypothesis (*P* < 0.05).

## Results

All breath-hold divers performed the requested protocol without any deviation from the proposed planning. We did not find any significant difference in depth, diving time, bottom time, surface interval, and GF among the 20 divers.

We found a statistically significant glycemia decrease after dives, mean 94.3 ± 11.6 vs. 83.5 ± 12.5 (− 10.8%) *p* = 0.001; we also found a statistically significant increase of insulinemia median 4.5 range 3.4/6.4 vs. 7.0 range 4.2/10.2 (+ 51.5%) *P* < 0.0001 and a statistically significant urine catecholamine increase median 14.0 range 8/18 vs. 15.5 range 10.0/21.0 (+ 24.4%) *P* < 0.0001 (Table 1).

**Table 1:** Summary of clinical data

Clinical data		Before	After	Δ		*P* = value
Glycemia	Means	94.3 ± 11.6	83.5 ± 12.5	10.5	mg/dl	*P* = 0.001
Insulin	Median	4.5 range 3.4/6.4	7.0 range 4.2/10.2	2.3	mcgU/ml	*P* < 0.0001
Catecholamine	Median	14.0 range 8/18	15.5 range 10.0/21.0	3.1	μg	*P* < 0.0001

## Discussion

Our data show a decrease in glycemia and an increase in blood insulin after repetitive BH-diving (Additional file 1).

This metabolic response is typical in cases of intermittent hypoxia when an insulin-stimulated glucose uptake occurs [8]. This data also agree with Clanton’s manuscript that shows how glucose cellular uptake increases because hypoxia induces upregulation of HIF-1 [9].

The role of HIF-1α both in the insulin-mediated glucose uptake and in the expression of the GLUT-4 on the cell membrane is well known [9].

The increase of insulinemia during BH-diving associated with the decrease of glycemia could indicate that the upregulating cellular uptake is not caused by activation of the specific glucose transporters GLUT4, even if the complex changes caused by hyperbaric exposure do not permit to exclude that other mechanisms are involved in addition to the pilot role of insulin.

We also found an increase of urine catecholamine levels during the dives, while we would have expected that the level remained unchanged or decreased, considering that the participants were very expert BH-diving instructors and we did not expect any significant stress effect during the tests. The protocol requested, in fact, was not very hard, both for mental and physical aspects, considering the high experience level of the participants.

Even if BH-diving implies hyperbaric and consequently hyperoxic exposure, it should be remembered that during BH-diving, a mass of blood pools in the lungs as a result of the so-called blood-shift effect [2, 3]. This blood shifts from other districts that can tolerate moderate levels of hypo-perfusion without damage, such as the abdominal vessels, spleen, and striated muscle [3, 11]. This condition could produce a moderate muscle hypoxia and justify the insulin-stimulated glucose uptake.

This particular BH-diving-related condition could determine different levels of oxygenation in different organs, some being in normal conditions and others in a relative hypoxia state; we can therefore infer that the general scenario could be quite complex and different mechanisms for glucose transport could occur simultaneously [12].

On the other hand, this binary aspect could justify a different cell response and explain our conflicting data about the urine catecholamine increase that could be also explained by the need of peripheral vasoconstriction.

Our data showed that insulin-stimulated glucose uptake is involved in cellular metabolism during breath-hold diving but that other complex mechanisms may also be involved and need further investigation.

We can conclude that BH-diving is not a common exercise. All data seem to indicate that the observed changes are related to the diving reflex emphasized by the combination of cold-water face contact, exposure to pressure, and hypoxia.

A new specific protocol to investigate these aspects is in progress.

## Conclusions

The increase of insulin blood value during breath-hold diving associated with the decrease of glycemia confirms that the upregulating cellular uptake is not caused by activation of the specific glucose transporters GLUT4.

Our data confirm that the adaptations to BH-diving are caused by complex mechanisms and involve many largely unknown hormonal responses.

## Additional file

Additional file 1: Original data glucose uptake. (XLS 34 kb)

## Abbreviations

BH-divers: Breath-hold divers
BH-diving: Breath-hold diving
BMI: Body mass index
GF: Gradient factor
GLUT4: Glucose transporter type 4
HIF-1: Hypoxia-inducible transcription factor 1
SCUBA: Self-contained underwater breathing apparatus
